# Determining a healthy reference range and factors potentially influencing PRO-C3 – A biomarker of liver fibrosis

**DOI:** 10.1016/j.jhepr.2021.100317

**Published:** 2021-07-10

**Authors:** Elisabeth Erhardtsen, Daniel G.K. Rasmussen, Peder Frederiksen, Diana Julie Leeming, Diane Shevell, Lise Lotte Gluud, Morten Asser Karsdal, Guruprasad P. Aithal, Jörn M. Schattenberg

**Affiliations:** 1 Nordic Bioscience Biomarkers and Research, Herlev, Denmark; 2 Innovative Medicine, Bristol Myers-Squibb, Princeton, NJ, USA; 3 Department of Clinical Medicine, Faculty of Health and Medical Sciences, University of Copenhagen, Copenhagen, Denmark; 4 Gastro Unit, Medical Division, Hvidovre Hospital, University of Copenhagen, Hvidovre, Denmark; 5 Nottingham Digestive Diseases Centre, University of Nottingham, Nottingham, UK; 6 NIHR Nottingham Biomedical Research Centre at the Nottingham University Hospitals NHS Trust and the University of Nottingham, Nottingham, UK; 7 Medical Research Council (MRC), Nottingham Molecular Pathology Node, University of Nottingham, Nottingham, UK; 8 Department of Internal Medicine I, University Medical Centre of the Johannes Gutenberg-University, Mainz, Germany

**Keywords:** Humans, Non-alcoholic fatty liver disease, Collagen type III, Reference values, Ethnic groups, Healthy volunteers, Body mass index, Fibrosis, Biomarkers, Biopsy, Reference standards, Hospitals, ADAM, A Disintegrin and Metalloproteases, ALP, alkaline phosphatase, ALT, alanine aminotransferase, AST, aspartate aminotransferase, AUROC, area under the receiver operating characteristics curve, CLSI, Clinical and Laboratory Standards Institute, ELF™ test, Enhanced Liver Fibrosis test, FIB-4, fibrosis-4, LITMUS, Liver Investigation: Testing Marker Utility in Steatohepatitis (consortium), NAFLD, non-alcoholic fatty liver disease, NAS, NAFLD Activity Score, NASH, non-alcoholic steatohepatitis, NASH-CRN, NASH Clinical Research Network, NIMBLE, Non-Invasive Biomarkers of Metabolic Liver Disease (consortium), NPV, negative predictive value, PPV, positive predictive value, T2DM, type 2 diabetes mellitus

## Abstract

**Background & Aims:**

Progressive fibrosis has been identified as the major predictor of mortality in patients with non-alcoholic fatty liver disease (NAFLD). Several biomarkers are currently being evaluated for their ability to substitute the liver biopsy as the reference standard. Recent clinical studies in NAFLD/NASH patients support the utility of PRO-C3, a marker of type III collagen formation, as a marker for the degree of fibrosis, disease activity, and effect of treatment. Here we establish the healthy reference range, optimal sample handling conditions for both short- and long-term serum storage, and robustness for the PRO-C3 assay.

**Methods:**

PRO-C3 was measured in 269 healthy volunteers and in 222 NAFLD patients. Robustness of the PRO-C3 assay was measured according to Clinical and Laboratory Standards Institute standards and included validation of interference, precision, and reagent stability, whilst sample stability was defined for storage at different temperatures and for 3 freeze-thaw cycles. Fibrosis scoring was based on histological assessments and used as a reference for the diagnostic ability of PRO-C3 to discriminate between patients with different levels of fibrosis.

**Results:**

Robustness of the PRO-C3 analysis validated by interference, precision, and reagent stability was found to be within the predefined acceptance criteria. The healthy reference range was determined to be 6.1–14.7 ng/ml. Levels of PRO-C3 were not affected by sex, age, BMI, or ethnicity. Levels of PRO-C3 were able to identify patients with clinically significant fibrosis and advanced fibrosis (AUC = 0.83 (95% CI [0.77–0.88], p <0.0001), and AUC = 0.79 (95% CI [0.73–0.85], p <0.0001), respectively).

**Conclusions:**

The assay proved to be robust and sample stability was found to comply with hospital sample handling requirements. PRO-C3 measured in samples from patients with NAFLD/NASH was diagnostic for significant and advanced liver fibrosis.

**Lay summary:**

We showed that PRO-C3 levels were stable under conditions conforming with hospital sample-handling requirements. We determined a healthy reference range and showed that PRO-C3 levels were not associated with sex, age, BMI, or ethnicity. Finally, we provide further evidence of an association of PRO-C3 with increasing liver fibrosis.

## Highlights

• A normal reference range in healthy adult volunteers was established for PRO-C3.
• PRO-C3 levels were not affected by age, sex, BMI, or ethnicity.
• PRO-C3 levels increased with liver fibrosis stage.
• PRO-C3 identified patients with significant and advanced liver fibrosis.

## Graphical abstract

**Figure:**
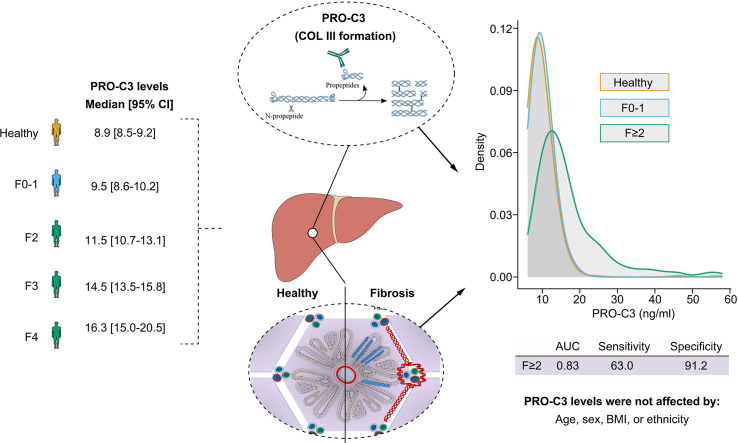

## Introduction

Non-alcoholic fatty liver disease (NAFLD) is a common progressive disorder often associated with the clinical features of metabolic syndrome. NAFLD starts with an excessive deposition of fat within hepatocytes for reasons other than excessive alcohol intake, especially obesity and type 2 diabetes mellitus (T2DM).^1^^,^^2^ The condition encompasses a wide spectrum ranging from isolated steatosis and non-alcoholic steatohepatitis (NASH), a condition with additional inflammation and hepatocyte injury. NASH can progress through varying degrees of fibrosis to cirrhosis, liver decompensation, and potentially hepatocellular carcinoma.^3^^,^^4^ In parallel with the worldwide increase in obesity and T2DM, NAFLD prevalence is also increasing.[5], [6], [7] With a global prevalence of 25%, NAFLD is now the leading cause of chronic liver disease in the USA and Europe.^8^^,^^9^ Although simple steatosis does not correlate with increased short-term morbidity or mortality, NAFLD patients can progress to include liver cell damage (NASH) and may also have fibrosis.^10^^,^^11^ Because of the dependence of histological features for a diagnosis of NASH and the necessity of invasive liver-biopsy confirmation, the exact prevalence of NASH in the general population is currently unknown. However, studies have shown that between 7 and 30% of NAFLD patients undergoing voluntary liver biopsies had NASH, and that the overall prevalence of NASH was between 1.5 and 6.45%.^6^

Despite several efforts to identify a non-invasive diagnostic method, liver biopsy remains the established, but imperfect, reference standard for definitive diagnosis of the spectrum of NAFLD disease.^12^^,^^13^ It is invasive, resource intensive, prone to sampling error and carries a small but significant risk of complications such as bleeding and pain.^14^ Participants in ongoing NASH drug trials are required to be staged by liver biopsy to qualify for enrolment.^15^^,^^16^ During the enrolment many patients undergoing liver biopsy do not meet the prespecified histological requirements and fail the screening process, having undergone an invasive procedure. Consequently, there is an urgent need for regulatory approved diagnostic biomarkers to facilitate the evaluation of new drugs. PRO-C3 is a type III collagen marker reflecting levels of the N-terminal pro-peptide released by A Disintegrin and Metalloproteases (ADAM)-TS2 during collagen maturation.^17^ The assay specifically detects the neo-epitope when the pro-peptide is released. In clinical NAFLD studies, PRO-C3 has been shown to be correlated with the fibrosis stage and additionally to be related to disease activity.[18], [19], [20] Furthermore, the anti-fibrotic efficacy of a drug in patients responding to therapy has been shown to correlate to serologically assessed PRO-C3.^21^ In the current study we established a healthy reference range, tested the optimal sample handling conditions for short- and long-term serum storage, and investigated the diagnostic ability of PRO-C3 to detect the histological fibrosis stage.

## Materials and methods

### Study groups

All samples used to determine the reference range and the technical validations included in the analysis for assay robustness were collected from apparently healthy individuals (healthy volunteers) and NAFLD patients. All individuals gave their written informed consent. The study protocols were approved by appropriate ethics committees, assigned for the individual hospitals (Hvidovre hospital) or clinics (Discovery Life Sciences and Reprocell). The NAFLD/NASH diagnosis was based on a liver biopsy. Liver biopsy was not available for healthy adult volunteers with samples available (obtained from Discovery Life Sciences and Reprocell).

Serum samples from 269 healthy adult volunteers representative of the US population in age, race, and sex were obtained from Discovery Life Sciences. All tested negative for chronic hepatitis. Of these, 101 were considered overweight (BMI 25.0–29.5 kg/m^2^) and 85 considered obese (BMI above 30). All volunteers were determined to be healthy after annual health checks. Volunteers with a history of diabetes or liver diseases or abnormal values of alanine aminotransferase (ALT; >50 IU/L), aspartate aminotransferase (AST; >50 IU/L), alkaline phosphatase (ALP; >129 IU/L), or bilirubin (>22 μmol/L) were excluded from the reference range estimation.

For the evaluation of PRO-C3 levels in NAFLD patients, a total of 222 patients with NAFLD were included from Nottingham University Hospitals NHS Trust (n = 83; Cohort 1) and the University Medical Center Mainz (n = 139; Cohort 2).^22^^,^^23^ All patients were informed about the rationale and possible risks of the study and provided their informed consent. The study protocols were approved by the Nottingham University Hospitals NHS Trust and the Ethikkommision of the Landesärztekammer Rheinland-Pfalz (No. 837.199'10 [7208]). Based on a liver biopsy taken within 3 months of obtaining blood samples, histological activities of inflammation and fibrosis were assessed for all patients. The grade of NASH was assessed by an experienced histopathologist using the NAFLD activity score (NAS) scored from 0 to 8, incorporating scores of steatoses (0–3), ballooning (0–2), and lobular inflammation (0–3). The NASH Clinical Research Network (NASH-CRN) grading and staging system was used for quantification of fibrosis.^13^

### Biochemical measurements

PRO-C3 was assessed using a competitive ELISA developed and produced at Nordic Bioscience (Herlev, Denmark). The assay procedure was as previously described.^17^ The PRO-C3 assay used a monoclonal antibody detecting the sequence CPTGPQNYSP (Nordic Bioscience), corresponding to the cleavage site of the pro-peptide from the mature collagen in position 153 of the collagen type III α1 chain. Each analytical run was calibrated by an 8-point standard curve measured in duplicates and 3 quality controls were included in duplicates per analytical run. Data below the limit of quantification were reported as the LLOQ value.

Biochemical measurements such as ALT, AST, ALP, and other variables were measured on validated platforms in central laboratories at the respective clinical centres: Nottingham University Hospitals NHS and the University Medical Center Mainz. The biochemical measurements of ALT, AST, ALP, and bilirubin in all healthy volunteers included in the reference range study were measured on validated platforms in a Clinical Laboratory Improvement Amendments accredited laboratory.

### Technical validation

To determine robustness of the PRO-C3 assay, we tested the assay according to available standards.[24], [25], [26], [27], [28] The PRO-C3 assay was tested in the following categories: analyte stability (storage and freeze-thaw), reagent stability, interference from known endogenous and the most relevant exogenous compounds used in treating patients with NAFLD/NASH, and assay precision. The test conditions, number of lots, operators, samples, and minimal acceptance criteria for each of the tests are summarised in Table 1.

**Table 1:** **Technical validation**.

Test	Conditions tested	Number of 1) Lots 2) Operators 3) Samples	Minimal acceptance criteria	Reference
Analyte stability – storage	Samples were stored at -80, 8, and 25°C for up to 14 months. Samples measured at 24 , 48 , 72 h, 8 days, 1, 3, 6, 12, and 24 months	1) 1 2) 1 3) 10	RE% ≤10% from nominal concentration and a weighted deeming slope of 1.0±0	Guideline on bioanalytical method validation^24^
Analyte stability – freeze-thaw	Samples tested for 3 freeze-thaw cycles	1) 1 2) 1 3) 10	RE% ≤10% from nominal concentration and a weighted deeming slope of 1.0±0	Guideline on bioanalytical method validation^24^
Reagent stability	Samples tested at 0, 3, 6, 9, 12, and 14 months. Samples stored at -80°C. Timepoint 0	1) 1 2) 1 3) 3 internal controls (kit calibrator and 2 controls), and 10 samples	RE% ≤10% from nominal concentration and a weighted deeming slope of 1.0±0	CLSI EP25^25^ and BS EN ISO 23640:2015^26^
Interference	Endogenous and exogenous substances were tested at low and high concentrations according to recommendations	1) 1 2) 1 3) 1 low (10–12 ng/ml) and 1 high (20–25 ng/ml)	RE% ≤10% from nominal concentration	CLSI EP7-A2^27^
Precision	Patients samples were used to generate 6 pools of PRO-C3 concentrations covering the measurement range. The study was performed on 2 reagent lots by 2 different operators (operators were swapped between reagent lots every day) along 20 non-consecutive days.	1) 2 2) 2 3) 6	For each sample: CV% ≤10% within 1 run Between-operator reproducibility: for each sample CV% ≤15%. Lot-to-lot variability: for each sample CV% ≤15% between reagent lots. Total precision: For each sample CV% ≤15%.	CLSI EP05-A3^28^

All validations were performed in serum samples from non-alcoholic fatty liver disease patients. CLSI, Clinical and Laboratory Standards Institute; CV%, coefficient of variation; RE%, percent recovery.

### Statistics

To test for differences between 2 groups, we used a Mann-Whitney test. For multiple groups, we used a Kruskal-Wallis test followed by Dunn’s test with correction for multiple comparisons.

The reference range for PRO-C3 was determined according to the Clinical and Laboratory Standards Institute standard EP28-A3c.^29^ The data distribution was evaluated by density plots and boxplots, both for the reference range and the NAFLD/NASH cohort. Outliers were detected on log-transformed data overall or within strata of sex, age, race, or obesity using Tukey’s criterion, and excluded before the reference range estimation. The reference range was estimated using the robust method on log-transformed data, and back transformed to the original scale of PRO-C3. Ninety percent CI estimates for the lower and the upper limit of the estimated reference were calculated using 5,000 bootstrap replicates of the data.

Outliers for the reference intervals of PRO-C3 in patients with NAFLD/NASH were detected on log-transformed data within strata of fibrosis stage, NASH, or fibrotic NASH using Tukey’s criterion, and excluded before the reference interval estimation. Area under receiving operator characteristic (AUROC) analyses were used to explore the diagnostic accuracy of PRO-C3 with respect to fibrosis staging in the NAFLD/NASH cohort. Ninety-five percent CI estimates for the estimated AUCs were calculated by the method of DeLong *et al.*^30^ Optimal cut-offs were estimated by maximising the Youden index.

The analyses were performed in R version 4.0.3 (R Foundation for Statistical Computing, Vienna, Austria).^31^ Reference ranges were estimated using the referenceIntervals package,^32^ and AUROC analyses were performed using the pROC package.^33^

## Results

### Technical validation

The serum PRO-C3 analyte was found to be stable for 8 days when stored at 8°C (Fig. S1A), for up to 48 h when stored at 25°C for 24 h (Fig. S1B), and remained stable up to the maximum time tested when stored at -80°C (Fig. S1C). PRO-C3 remained stable for all 3 freeze-thaw cycles tested (Fig. S1D). The PRO-C3 ELISA passed all predefined acceptance criteria for precision (intra- and interassay variation, lot variation, and interobserver variations (Table S1).

To test interference of potential endogenous and exogenous compounds (medication), the highest expected concentrations to be observed in patients’ circulation were spiked into serum samples from patients with NASH that either contained low or high PRO-C3 levels (Table S2). No interference was detected from the endogenous and exogenous compounds that were deemed most relevant for patients with NAFLD (Table S1).

### Reference ranges

**Fig. 1:**
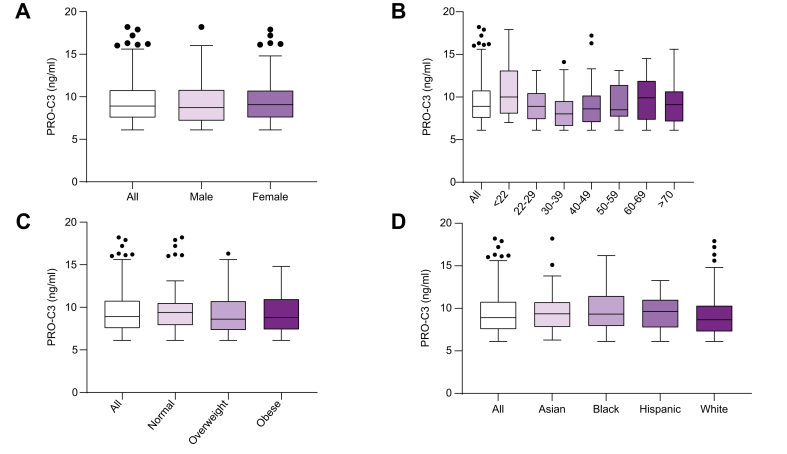
PRO-C3 levels in healthy volunteers. PRO-C3 levels were measured in 269 healthy volunteers. PRO-C3 levels were stratified based on (A) sex, (B) age, (C) BMI categories (healthy [BMI 18.5–24.9], overweight [BMI 25.0–29.5], and obese [BMI ≥30]), and (D) race. Data were plotted using the Tukey’s method. The Tukey’s whiskers reflect 1.5 times the IQR (25th to 75th percentile) or the highest or lowest datapoint, whichever is shorter. Differences between sex were determined based on a Mann-Whitney test. Differences between multiple groups were determined using a Kruskal-Wallis test. No significant differences were observed between the investigated groups.

Reference ranges for healthy volunteers were based on 269 apparently healthy individuals with no prior history of diabetes, liver disease, and with levels of ALT ≤50 IU/L, AST ≤50 IU/L, ALP ≤129 IU/L, or bilirubin ≤22 μmol/L were considered to be normal. The reference intervals for PRO-C3 in healthy individuals stratified by sex, age, BMI categories, and race are listed in Table 2. When comparing levels of PRO-C3 in the different stratifications, we saw that there was no significant difference in PRO-C3 levels between healthy males and females (Fig. 1A), based on age (Fig. 1B), BMI categories (Fig. 1C) or ethnicity (Fig. 1D).

**Table 2:** **PRO-C3 reference range in healthy volunteers**.

	N	Lower limit (ng/ml) [90% CI]	Upper limit (ng/ml) [90% CI]	Median (ng/ml)	Skewness
Total healthy	269	6.1 [6.1–6.1]	14.7 [14.0–15.3]	8.9 [8.5–9.2]	0.36∗
Sex					
Male	103	6.1 [6.1–6.1]	14.9 [13.6–16.1]	8.7 [8.4–9.1]	0.45
Female	166	6.1 [6.1–6.1]	14.6 [13.9–15.5]	9.1 [8.5–9.6]	0.3
Age, years					
≤22	13	6.1 [6.1–6.5]	20.0 [15.0–25.3]	10.0 [8.5–13.1]	0.35
22–29	58	6.1 [6.1–6.1]	14.6 [13.3–16.0]	9.1 [8.4–9.7]	0.22
30–39	64	6.1 [6.1–6.1]	13.6 [12.5–14.8]	8.5 [7.9–9.5]	0.37
40–49	44	6.1 [6.1–6.1]	14.3 [12.6–16.0]	8.4 [8.0–9.3]	0.5
50–59	46	6.1 [6.1–6.1]	15.1 [13.2–16.8]	8.6 [7.9–9.6]	0.41
60–69	26	6.1 [6.1–6.9]	16.9 [14.7–19.1]	10.1 [8.6–11.1]	-0.22
70+	17	6.1 [6.1–6.1]	16.0 [13.2–19.3]	9.1 [7.3–10.5]	0.27
BMI stage					
Normal	69	6.1 [6.1–6.2]	14.8 [13.6–16.0]	9.4 [8.5–9.8]	0.22
Overweight	101	6.1 [6.1–6.1]	14.1 [13.1–15.2]	8.5 [8.1–8.9]	0.39
Obese	96	6.1 [6.1–6.1	14.6 [13.6–15.5]	9.0 [8.4–9.3]	0.15
Ethnicity					
Asian	27	6.1 [6.1–6.6]	14.5 [12.8–16.5]	9.3 [8.2–10.4]	0.32
Black	51	6.1 [6.1–6.1]	16.1 [14.5–17.9]	9.3 [8.4–10.6]	0.25
Hispanic	22	6.1 [6.1–6.9]	15.1 [13.2–16.8]	9.6 [8.4–10.4]	-0.34
White	167	6.1 [6.1–6.1]	14.1 [13.3–14.9]	8.6 [8.1–9.0]	0.36

Stratified according to sex, age strata, obesity, and ethnicity. BMI status was defined as healthy (BMI 18.5–24.9), overweight (BMI 25.0–29.5), and obese (BMI ≥30). Except for the age groups ‘<22’ and ‘70+’ in which the number of patients was too low, the lower and upper limits with 90% CIs were estimated using the robust method (CLSI C28-A3). For the age group ‘≤20’ and ‘>70’ the lower and upper limits were estimated using the robust method, but the 90% CIs for the lower and upper limits were estimated based on the assumption that the data had a log-normal distribution. PRO-C3 levels below the lower limit of quantification were assigned the lowest acceptable concentration. Outliers were detected on log-transformed data using test Tukey’s criterion, and excluded. Skewness estimates are based on the log-transformed data. Significance level: ∗*p* <0.05.

### PRO-C3 values in NASH patients

**Fig. 2:**
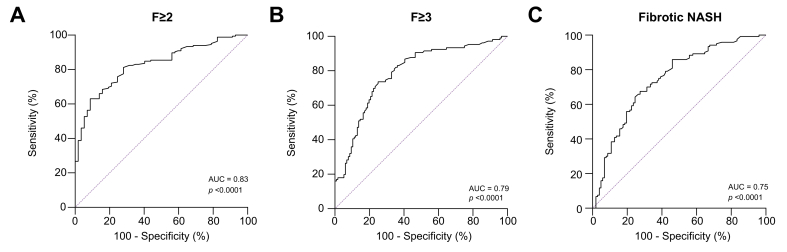
Diagnostic ability of PRO-C3. We investigated the ability of PRO-C3 as a diagnostic screen tool for patients with (A) clinically significant and (B) advanced fibrosis, as well as (C) fibrotic NASH. We defined clinically significant fibrosis as patients having NASH with fibrosis stage ≥F2. Fibrotic NASH was defined as lobular inflammation ≥1, ballooning ≥1, and NAS ≥4, and fibrosis stage ≥F2. Figures were generated using area under the receiver operating characteristics curve analysis. NAS, non-alcoholic fatty liver disease activity score; NASH, non-alcoholic steatohepatitis.

Next, we investigated levels of PRO-C3 in 215 patients (7 outliers were excluded from the analysis) with NAFLD/NASH (Table 3). Patients had a mean (±SD) age of 56 (46.6–63.3), and 51.8% were male, demographic data are listed in Table S3. Of the investigated patients, 56 (26%) had no or mild fibrosis (F0–F1), 59 (27 %) had intermediate fibrosis (F2), 60 (28%) had advanced fibrosis (F3), and 40 (19%) had cirrhosis (F4). Based on histological findings, 134 (62%) patients had NASH (*i.e.* lobular inflammation ≥1, ballooning ≥1, and NAS ≥4) and 119 (55%) had fibrotic NASH (*i.e.* NASH with fibrosis stage ≥2). As described previously, levels of PRO-C3 increased with the fibrosis stage (Table 3; *p* <0.0001). Next, we investigated the diagnostic ability of PRO-C3 to discriminate between patients with no or mild fibrosis and patients with significant fibrosis (≥F2). Levels of PRO-C3 were able to discriminate between these groups with an AUC = 0.83 (95% CI [0.77–0.88], sensitivity = 63.0%, specificity = 91.2%, positive predictive value (PPV) = 95.4%, negative predictive value (NPV) = 46.0%, *p* <0.0001; Fig. 2A and Table 4). Levels of PRO-C3 were able to discriminate between patients with no, mild, or intermediate fibrosis, and patients with advanced fibrosis (≥F3) with an AUC = 0.79 (95% CI [0.73–0.85], sensitivity = 73.6%, specificity = 75.0%, PPV = 72.9%, NPV = 75.7%, *p* <0.0001; Fig. 2B and Table 4). Based on guidance documents from the regulatory authorities, the recommended patients to be enrolled in interventional clinical trials are NASH patients with fibrosis stage 2 and 3.^16^^,^^34^ To assess the utility of PRO-C3 as a screening tool we investigated the ability to identify NASH patients with clinically significant fibrosis NASH (fibrotic NASH). Specifically, clinically significant fibrotic NASH defined as lobular inflammation ≥1, ballooning ≥1, NAS ≥4, and fibrosis stage ≥2. PRO-C3 was able to discriminate patients without clinically significant fibrosis and NASH patients with clinically significant fibrosis (fibrotic NASH) with an AUC = 0.75 (95% CI [0.68–0.81], sensitivity = 67.5%, specificity = 72.5%, PPV = 74.3%, NPV = 65.5%, *p* <0.0001; Fig. 2C and Table 4).

**Table 3:** **Reference intervals of PRO-C3 in patients with NAFLD or NASH**.

	N	Lower limit (ng/ml) [90% CI]	Upper limit (ng/ml) [90% CI]	Median (ng/ml) [90% CI]	Skewness
F0/F1	56	6.1 [6.1–6.4]	15.0 [13.7–16.2]	9.5 [8.6–10.2]	0.019
F2	59	6.1 [6.1–6.5]	25.4 [21.2–29.5]	11.5 [10.7–13.1]	0.63∗
F3	60	8.0 [7.1–9.1]	27.4 [24.1–30.7]	14.5 [13.5–15.8]	-0.49
F4	40	6.1 [6.1–7.3]	54.2 [39.1–69.8]	16.3 [15.0–20.5]	0.54
NASH	134	6.3 [6.1–6.9]	31.2 [27.8–35.0]	13.8 [12.9–14.9]	0.47∗
Fibrotic NASH	119	6.5 [6.1–7.2]	34.4 [30.0–38.9]	14.6 [13.7–15.9]	0.56∗

Reference intervals were estimated by the robust method according to the recommended approach (CLSI C28-A3). PRO-C3 levels below the lower limit of quantification were assigned the lowest acceptable concentration. NASH was defined as lobular inflammation ≥1, ballooning ≥1, and NAS ≥4. Fibrotic NASH was defined as NASH with fibrosis stage ≥2. Outliers were detected on log-transformed data using test Tukey’s criterion, and excluded. Skewness estimates are based on the log-transformed data. Statistical significance: ∗*p* <0.05. CLSI, Clinical and Laboratory Standards Institute; NAS, non-alcoholic fatty liver disease activity score; NASH, non-alcoholic steatohepatitis.

**Table 4:** **Ability of PRO-C3 to distinguish between relevant subgroups of NAFLD/NASH patients**.

	AUC [95% CI]	Cut-off [95% CI]	Sensitivity (%)	Specificity (%)	PPV (%)	NPV (%)	*p* value
F ≥2	0.83 [0.77–0.88]	12.6 [12.0–14.8]	63.0	91.2	95.4	46.0	<0.0001
F ≥3	0.79 [0.73–0.85]	12.7 [10.9–15.3]	73.6	75.0	72.9	75.7	<0.0001
Fibrotic NASH	0.75 [0.68–0.81]	12.6 [10.5–15.5]	67.5	72.5	74.3	65.5	<0.0001

Estimated AUCs and cut-off values for the identification of patients with F ≥2, F ≥3, and fibrotic NASH (NASH with F ≥2) are listed along with sensitivity, specificity, positive predictive value (PPV), and negative predictive value (NPV). Curves were generated using area under the receiver operating characteristics curve analysis. The 95% CIs for the AUC and cut-offs were estimated by bootstrapping.

## Discussion

Several biomarkers have been developed for NAFLD in recent years, including surrogate risk scores, individual blood-based markers, complex panels, and new imaging modalities.^12^^,^^35^ PRO-C3 is a relatively new biomarker and is detected by an ELISA that specifically targets the neo-epitope of the N-terminal pro-peptide of type III collagen, which is released during deposition of the fibrillar collagen in the extracellular matrix. In liver fibrosis, type III collagen production is markedly upregulated. During its formation and accumulation pro-peptides are released from type III pro-collagen by ADAM-TS2-mediated cleavage and released into the circulation.^36^ Consequently, the PRO-C3 ELISA measures a fragment reflecting active fibrogenesis.

Several studies have explored PRO-C3 within clinical trials.^15^^,^^18^^,^^19^^,^^21^^,^^23^^,^[36], [37], [38], [39], [40], [41], [42] A recent meta-analysis included studies that provided data on biopsy-proven NAFLD, PRO-C3 test, and data on true positive, false positive, true negative, and false negative results, or data that allowed a reconstruction of the classification table, were eligible for inclusion in the meta-analysis (submitted). Five studies consisting of 1,312 patients reported data on significant fibrosis with thresholds ranging from 13.2 to 21.3 ng/ml.^15^^,^^18^^,^^23^^,^^38^^,^^39^ Two of the studies included data on PRO-C3 in detecting (fibrotic) NAFLD/NASH and found thresholds of 14.5 and 14.7 ng/ml, respectively.^15^^,^^39^ For cirrhosis related to NAFLD/NASH thresholds of 15.6 and 16.5 ng/ml have previously been reported.^15^^,^^23^ Our findings seem to be in line with previous findings.

To support further clinical investigations and use of PRO-C3 it is critical to establish robustness of the analysis, optimal sample handling, conditions for short- and long-term serum storage, and a healthy reference range. In this article we have proven the assay to be robust, including precision within expected limits, shown that the analyte and reagents are stable for periods that are in alignment with clinical use, and not influenced by potential endogenous and exogenous (medications) which are highly relevant for the NAFLD population. However, as the NAFLD population includes patients who often suffer from accompanying diseases, it may be beneficial to test whether medications of selected co-morbidities may influence PRO-C3 levels.

Based on our findings in healthy individuals, we showed that PRO-C3 levels were not influenced by sex, age, or race. This finding contrasts with the Enhanced Liver Fibrosis (ELF™) test which has been found to be significantly dependent on age and sex, leading the authors to stress that this score needs to be interpreted with caution.^43^ Importantly, being overweight or obese – a major risk factor for NAFLD did not impact PRO-C3. Serum PRO-C3 values were not associated with BMI, and levels of PRO-C3 were similar in patients classified as normal weight (BMI 18.5–24.9), overweight (BMI 25.0–29.5), and obese (BMI ≥30).

At current, regulatory agencies (FDA and EMA) require phase IIb and phase III clinical trials to report NASH-resolution or fibrosis regression based on liver biopsy results in order to consider conditional approval for a drug. This is a major limitation for drug development as recruitment rates are impacted and thereby major delays in the development timelines for a specific compound emerge. Large-scale efforts are currently ongoing by 2 major consortia, namely the European consortium LITMUS (Liver Investigation: Testing Marker Utility in Steatohepatitis)^44^ and the US consortium NIMBLE (Non-Invasive Biomarkers of Metabolic Liver Disease),^45^ to define what the FDA and EMA requires for a biomarker qualification (*e.g.* level of technical, clinical, statistical analysis) in different contexts of uses in NAFLD/NASH. A qualification of a diagnostic biomarker could ultimately amend or even eliminate the need for a liver biopsy for patients entering clinical trials, if robust data are available.

We next evaluated levels of PRO-C3 in a cohort of NAFLD patients with histological assessments available. The NAFLD patient group consisted of cohorts from 2 centres in Germany and the UK. We showed that levels of PRO-C3 were able to discriminate patients with no or mild fibrosis from patients with significant fibrosis (≥F2). We also tested the performance of PRO-C3 for specifically identifying advanced fibrosis, as well as fibrotic NASH. The performance of PRO-C3 to discriminate these patients proved to be slightly lower than the discrimination of patients with significant fibrosis; however, the AUROC was only slightly lower. The lower AUROC may be attributable to a relatively low number of patients in these 2 groups. Overall, the performance of PRO-C3 in our paper is in line with findings from a recent review and metanalysis of PRO-C3 in previous studies of patients with NAFLD/NASH (submitted).

The current data support the use of PRO-C3 as a diagnostic screening marker. Such a marker could be used to de-select patients that would be unlikely to meet histological enrolment criteria, and thereby avoid an unnecessary procedure with the potential for complications.

Another option for use of PRO-C3 is as a prognostic enrichment biomarker which could enrich a trial with patients who are more likely to develop adverse outcomes. However, for the EMA and FDA to qualify a prognostic biomarker for clinical events, they are likely to require real-time data on clinical endpoints, which will only be generated in large scale clinical studies exceeding to 5–8 years in duration considering the (in most cases) slowly progressive nature of fibrosis in NAFLD.

In recent studies, a combination of PRO-C3 with other clinical markers was shown to be a good diagnostic screening tool.^20^^,^^23^ The studies investigated the ability of a combination of different markers (PRO-C3 and other laboratory markers^23^) to identify patients meeting the recommended biopsy requirements by the FDA and EMA for phase III trial enrolment (*i.e.* NAS ≥4 with at least 1 point each in inflammation and ballooning along with a NASH-CRN >1 but < stage 4).^16^^,^^34^ The combination was suggested to be a possible means to decrease the number of patients to be biopsied, as patients with a low score would have a low probability of meeting the biopsy enrolment criteria and would undergo an unnecessary biopsy.

Biomarkers are needed in the management of NAFLD patients to: (i) determine severity of disease (staging), (ii) develop new treatments, and (iii) monitor patients to identify patients at risk of disease progression and adverse outcomes. In relation to determining disease severity, such markers may reduce the number of unnecessary biopsies performed for clinical trial enrolment.

Several clinical studies have evaluated and defined usage of PRO-C3 in the NAFLD/NASH population. This includes positive data on; the ability of PRO-C3 to determine severity of disease,^15^^,^^18^^,^^23^^,^^37^^,^^42^ its ability as a prognostic biomarker to enrich clinical trials to monitor treatment effect,^19^^,^^21^^,^^38^^,^^41^^,^^42^^,^^46^ and for determining disease activity.^20^ The fact that PRO-C3 is a marker that directly reflects fibrosis and fibrosis activity makes it suitable for use in clinical practice for the general practitioner to support/take decisions on referral of patients to specialised liver centres, and in specialised liver centres for support in decisions on patient management and prognosis – potentially even without the need for a biopsy. The advantage of using PRO-C3 for initial evaluation of liver fibrosis compared with fibrosis-4 (FIB-4) and NAFLD fibrosis score is that PRO-C3 is a direct marker of fibrosis in contrast to FIB-4 and the NAFLD fibrosis score, which are indirect composite scores of fibrosis that rather reflect liver disease. The markers included in these composites scores are clinical characteristics, which mostly are markers of liver function, thus their origin is not the liver scar tissue. The PRO-C3 and ELF score composite scores are direct markers of liver fibrosis, as they assess proteins which originate from scar tissue of the liver. Furthermore, in a study of more than 400 patients with NAFLD, it was shown that PRO-C3, as a single marker or as a composite marker within the ADAPT score (age, diabetes, platelets, and PRO-C3), was superior to APRI (AST to platelet ratio index), FIB-4, and NAFLD Fibrosis score for identification of advanced liver fibrosis.^23^ A similar analysis was performed in more than 300 patients supporting that ADAPT is superior.^47^ The ELF score requires the assessment of 3 fibrosis-related markers (PIIINP, HA, TIMP-1) in contrast to PRO-C3 which is a single marker. Nevertheless, it has been indicated that PRO-C3 is a more dynamic marker of fibrogenesis compared to ELF, shown in several clinical trial studies of NASH or primary sclerosing cholangitis (PSC), where reduction of PRO-C3 were greater compared with ELF and also the PIIINP component alone, during treatment of patients with potential anti-fibrotic therapies.^40^^,^^48^^,^^49^ This may suggest that PRO-C3 is a superior marker for liver fibrosis because of its dynamics and origin within liver fibrosis. Future studies will determine whether PRO-C3 also has a place in the general clinical management of NAFLD patients. However, it has already been shown that the serial combination of ADAPT with liver stiffness measurement has high diagnostic accuracy with a low requirement for liver biopsy.^47^ The proposed algorithm would help stratify those who need biopsies and narrow down those patients who would need to be referred to specialty clinics.

In apparently healthy volunteers resembling the US population we found that PRO-C3 levels were not associated with sex, age, BMI, or race. The assay was robust as measured in relation to interference, precision, reagent, and sample stability. Furthermore, we provided further evidence that PRO-C3 is a promising marker for pre-screening of patients for clinical trials to avoid unnecessary biopsies.

## Financial support

The biomarker validation studies were funded by 10.13039/100002491Bristol Myers-Squibb, Princeton, NJ, USA. The study was supported by the Danish Research Foundation.

## Authors’ contributions

In charge of the study concept and design: EE, DS, DGKR, JS

Acquired the data: EE, DJL, GA, JS

Analysed and interpreted the data: EE, PF, DGKR

Performed the statistical analysis: PF, DGKR

Drafted the manuscript: EE, DGKR

Critical revision of the manuscript for important intellectual content: all authors

## Data availability

Data are available upon request and an appropriate institutional collaboration agreement. Data are not available to access in a repository owing to concern that the identity of patients might be revealed inadvertently.

## Conflicts of interest

JS has acted as consultant to Boehringer Ingelheim, BMS, Echosens, Genfit, Gilead Sciences, Intercept Pharmaceuticals, Madrigal, Novartis, Novo Nordisk, Nordic Bioscience, Pfizer, Roche, Sanofi, Siemens Healthcare GmbH, Zydus, has received research funding from Gilead Sciences, and has acted as speaker for Falk Foundation MSD Sharp & Dohme GmbH. All outside the submitted work. EE, DGKR, PF, DJL, and MAK, are employees of Nordic Bioscience, a company engaged in the development of biochemical markers. MAK and DJL are stockholders of Nordic Bioscience. LLG has acted as consultant for Novo Nordisk and received funding for research from Novo Nordisk, Gilead, Alexion, and Vingmed, all outside the submitted work. DS is an employee of Bristol Myers Squibb. GA has associations with Shire, Agios, and GlaxoSmithKline, and advises the Medicines and Healthcare products Regulatory Agency (MHRA), all outside the submitted work.

## References

[1] Adams L.A., Anstee Q.M., Tilg H., Targher G. (2017). Non-alcoholic fatty liver disease and its relationship with cardiovascular disease and other extrahepatic diseases. Gut.

[2] Hadi A., Mohammadi H., Miraghajani M., Ghaedi E. (2019). Efficacy of synbiotic supplementation in patients with nonalcoholic fatty liver disease: a systematic review and meta-analysis of clinical trials: synbiotic supplementation and NAFLD. Crit Rev Food Sci Nutr.

[3] Anstee Q., Daly A., Day C. (2011). Genetics of alcoholic and nonalcoholic fatty liver disease. Semin Liver Dis.

[4] Anstee Q.M., Targher G., Day C.P. (2013). Progression of NAFLD to diabetes mellitus, cardiovascular disease or cirrhosis. Nat Rev Gastroenterol Hepatol.

[5] Wong V.W.-S., Chu W.C.-W., Wong G.L.-H., Chan R.S.-M., Chim A.M.-L., Ong A. (2012). Prevalence of non-alcoholic fatty liver disease and advanced fibrosis in Hong Kong Chinese: a population study using proton-magnetic resonance spectroscopy and transient elastography. Gut.

[6] Younossi Z., Anstee Q.M., Marietti M., Hardy T., Henry L., Eslam M. (2018). Global burden of NAFLD and NASH: trends, predictions, risk factors and prevention. Nat Rev Gastroenterol Hepatol.

[7] Vernon G., Baranova A., Younossi Z.M. (2011). Systematic review: the epidemiology and natural history of non-alcoholic fatty liver disease and non-alcoholic steatohepatitis in adults. Aliment Pharmacol Ther.

[8] Younossi Z.M., Stepanova M., Afendy M., Fang Y., Younossi Y., Mir H. (2011). Changes in the prevalence of the most common causes of chronic liver diseases in the United States from 1988 to 2008. Clin Gastroenterol Hepatol.

[9] Younossi Z.M., Koenig A.B., Abdelatif D., Fazel Y., Henry L., Wymer M. (2016). Global epidemiology of nonalcoholic fatty liver disease—meta-analytic assessment of prevalence, incidence, and outcomes. Hepatology.

[10] Singh S., Allen A.M., Wang Z., Prokop L.J., Murad M.H., Loomba R. (2015). Fibrosis progression in nonalcoholic fatty liver vs nonalcoholic steatohepatitis: a systematic review and meta-analysis of paired-biopsy studies. Clin Gastroenterol Hepatol.

[11] Chalasani N., Younossi Z., Lavine J.E., Charlton M., Cusi K., Rinella M. (2018). The diagnosis and management of nonalcoholic fatty liver disease: practice guidance from the American Association for the Study of Liver Diseases. Hepatology.

[12] Vilar-Gomez E., Chalasani N. (2018). Non-invasive assessment of non-alcoholic fatty liver disease: clinical prediction rules and blood-based biomarkers. J Hepatol.

[13] Kleiner D.E., Brunt E.M., Van Natta M., Behling C., Contos M.J., Cummings O.W. (2005). Design and validation of a histological scoring system for nonalcoholic fatty liver disease. Hepatology.

[14] Ratziu V., Charlotte F., Heurtier A., Gombert S., Giral P., Bruckert E. (2005). Sampling variability of liver biopsy in nonalcoholic fatty liver disease. Gastroenterology.

[15] Boyle M., Tiniakos D., Schattenberg J.M., Ratziu V., Bugianessi E., Petta S. (2019). Performance of the PRO-C3 collagen neo-epitope biomarker in non-alcoholic fatty liver disease. JHEP Rep.

[16] FDA/CDER (2018).

[17] Nielsen M.J., Nedergaard A.F., Sun S., Veidal S.S., Larsen L., Zheng Q. (2013). The neo-epitope specific PRO-C3 ELISA measures true formation of type III collagen associated with liver and muscle parameters. Am J Transl Res.

[18] Bril F., Leeming D.J., Karsdal M.A., Kalavalapalli S., Barb D., Lai J. (2019). Use of plasma fragments of propeptides of Type III, V, and VI procollagen for the detection of liver fibrosis in type 2 diabetes. Diabetes Care.

[19] Ratziu V., Sanyal A., Harrison S.A., Wong V.W.S., Francque S., Goodman Z. (2020). Cenicriviroc treatment for adults with nonalcoholic steatohepatitis and fibrosis: final analysis of the phase 2b CENTAUR study. Hepatology.

[20] Harrison S.A., Taub R.A., Karsdal M.A., Franc J., Bashi M., Barbone J.M. (2020). https://aasldpubs.onlinelibrary.wiley.com/doi/full/10.1002/hep.31579.

[21] Sanyal A., Charles E.D., Neuschwander-Tetri B.A., Loomba R., Harrison S.A., Abdelmalek M.F. (2019). Pegbelfermin (BMS-986036), a PEGylated fibroblast growth factor 21 analogue, in patients with non-alcoholic steatohepatitis: a randomised, double-blind, placebo-controlled, phase 2a trial. Lancet.

[22] Labenz C., Huber Y., Kalliga E., Nagel M., Ruckes C., Straub B.K. (2018). Predictors of advanced fibrosis in non-cirrhotic non-alcoholic fatty liver disease in Germany. Aliment Pharmacol Ther.

[23] Daniels S.J., Leeming D.J., Eslam M., Hashem A.M., Nielsen M.J., Krag A. (2019). ADAPT: an algorithm incorporating PRO-C3 accurately identifies patients with NAFLD and advanced fibrosis. Hepatology.

[24] (2012). Guideline on bioanalytical method validation.

[25] CLSI EP25-A (2009).

[26] BS EN ISO 23640:2015 In vitro diagnostic medical devices. Evaluation of stability of in vitro diagnostic reagents.

[27] CLSI EP07-A2 (2005).

[28] CLSI EP05-A3 (2014).

[29] (2010). CLSI C28-A3c Vol. 28 No. 30. Defining, Establishing and Verifying Reference Intervals in The Clinical Laboratory; Approved Guideline.

[30] DeLong E.R., DeLong D.M., Clarke-Pearson D.L. (1988). Comparing the areas under two or more correlated receiver operating characteristic curves: a nonparametric approach. Biometrics.

[31] Bunn A., Korpela M. (2020).

[32] Maintainer F., Finnegan D. (2020).

[33] Robin X., Turck N., Hainard A., Tiberti N., Lisacek F., Sanchez J.C. (2011). pROC: an open-source package for R and S+ to analyze and compare ROC curves. BMC Bioinformatics.

[34] EMA (2018). http://www.ema.europa.eu/contact.

[35] Wong V.W.S., Adams L.A., de Lédinghen V., Wong G.L.H., Sookoian S. (2018). Noninvasive biomarkers in NAFLD and NASH — current progress and future promise. Nat Rev Gastroenterol Hepatol.

[36] Karsdal M.A., Daniels S.J., Holm Nielsen S., Bager C., Rasmussen D.G.K., Loomba R. (2020). Collagen biology and non-invasive biomarkers of liver fibrosis. Liver Int.

[37] Luo Y., Oseini A., Gagnon R., Charles E.D., Sidik K., Vincent R. (2018). An evaluation of the collagen fragments related to fibrogenesis and fibrolysis in nonalcoholic steatohepatitis. Sci Rep.

[38] Huber Y., Pfirrmann D., Gebhardt I., Labenz C., Gehrke N., Straub B.K. (2019). Improvement of non-invasive markers of NAFLD from an individualised, web-based exercise program. Aliment Pharmacol Ther.

[39] Leeming D.J., Nielsen M.J., Goodman Z., Friedman S., Lefebvre E., Seyedkazemi S. (2017). Plasma collagen type III (PRO-C3) levels associate with severity of histological features of non-alcoholic steatohepatitis and fibrosis within the screening population from the CENTAUR study. NASH Biomarkers Work.

[40] Harrison S.A., Rossi S.J., Paredes A.H., Trotter J.F., Bashir M.R., Guy C.D. (2020). NGM282 improves liver fibrosis and histology in 12 weeks in patients with nonalcoholic steatohepatitis. Hepatology.

[41] Harrison S.A., Rinella M.E., Abdelmalek M.F., Trotter J.F., Paredes A.H., Arnold H.L. (2018). NGM282 for treatment of non-alcoholic steatohepatitis: a multicentre, randomised, double-blind, placebo-controlled, phase 2 trial. Lancet.

[42] Hartman M.L., Sanyal A.J., Loomba R., Wilson J.M., Nikooienejad A., Bray R. (2020). Effects of novel dual GIP and GLP-1 receptor agonist tirzepatide on biomarkers of nonalcoholic steatohepatitis in patients with type 2 diabetes. Diabetes Care.

[43] Lichtinghagen R., Pietsch D., Bantel H., Manns M.P., Brand K., Bahr M.J. (2013). The Enhanced Liver Fibrosis (ELF) score: normal values, influence factors and proposed cut-off values. J Hepatol.

[44] The Liver Investigation: Testing Marker Utility in Steatohepatitis (LITMUS) project. https://litmus-project.eu/. 10.1016/j.cct.2023.107352 37802221

[45] FNIH https://fnih.org/news/announcements/fnih-biomarkers-consortium-launches-nimble-replace-invasive-and-painful-biopsy.

[46] Harrison S.A., Bashir M.R., Guy C.D., Zhou R., Moylan C.A., Frias J.P. (2019). Resmetirom (MGL-3196) for the treatment of non-alcoholic steatohepatitis: a multicentre, randomised, double-blind, placebo-controlled, phase 2 trial. Lancet.

[47] Eslam M., Wong G.L.-H., Hashem A.M., Chan H.L.-Y., Nielsen M.J., Leeming D.J. (2021). A sequential algorithm combining ADAPT and liver stiffness can stage metabolic-associated fatty liver disease in hospital-based and primary care patients. Am J Gastroenterol.

[48] Hirschfield G.M., Chazouillères O., Drenth J.P., Thorburn D., Harrison S.A., Landis C.S. (2019). Effect of NGM282, an FGF19 analogue, in primary sclerosing cholangitis: a multicenter, randomized, double-blind, placebo-controlled phase II trial. J Hepatol.

[49] Harrison S.A., Neff G., Guy C.D., Bashir M.R., Paredes A.H., Frias J.P. (2021). Efficacy and safety of aldafermin, an engineered FGF19 analog, in a randomized, double-blind, placebo-controlled trial of patients with nonalcoholic steatohepatitis. Gastroenterology.

