# Supplementary material for: Determining a healthy reference range and factors potentially influencing PRO-C3 – A biomarker of liver fibrosis
Source: JHEP Rep. 2021 Jul 10;3(4):100317. doi: 10.1016/j.jhepr.2021.100317 (PMC8385245; doi:10.1016/j.jhepr.2021.100317)
Supplement: Multimedia component 1 [file mmc1.pdf]

Determining a healthy reference range and factors potentially influencing PRO-C3 – A biomarker of liver fibrosis

Elisabeth Erhardtsen, Daniel G. K. Rasmussen, Peder Frederiksen, Diana Julie Leeming, Diane Shevell, Lise Lotte Gluud, Morten Asser Karsdal, Guruprasad P. Aithal, Jörn M. Schattenberg

Table of contents

Supplementary methods 2

Fig. S1. 3

Table S1 4

Table S2 5

Table S3. 6

Supplementary references 7

Supplementary methods

To determine robustness of the PRO-C3 assay, we tested the assay according to available standards [1–5]. The PRO-C3 assay was tested in the following categories: analyte stability (storage and freeze-thaw), reagent stability, interference from known endogenous and the most relevant exogenous compounds used in treating patients with NAFLD/NASH, and assay precision. **Table 1** found in the main manuscript summarizes the test conditions, number of lots, operators, samples, and minimal acceptance criteria for each of tests. Acceptance criteria for analyte stability was defined as ≤10% change from fresh sample for each time point and a weighted Deeming slope of 1.0±0 when regressing the measurement from each condition to the fresh sample measurement.

For the evaluation of PRO-C3 levels in NAFLD patients, a total of 222 patients with NAFLD were included from Nottingham University Hospitals NHS Trust (n=83; Cohort 1) and the University Medical Center Mainz (n=139; Cohort 2) [6,7]. All patients were informed about the rationale and possible risks of the study and provided their informed consent. The study protocols were approved by the Nottingham University Hospitals NHS Trust and the Ethikkommision of the Landesärztekammer Rheinland-Pfalz (No. 837.199'10 (7208)). Based on a liver biopsy taken within 3 months of obtaining blood samples all patients' histological activity of inflammation and fibrosis were assessed. The grade of NASH was assessed by an experienced histopathologist using the NAFLD activity score (NAS) scored from (0-8), incorporating scores of steatoses (0-3), ballooning (0-2), and lobular inflammation (0-3). The NASH-CRN grading and staging system was used for quantification of fibrosis [8]. To test for differences between the two cohorts and the combined cohort, we used a Kruskal-Wallis test.

Fig. S1. Analyte stability

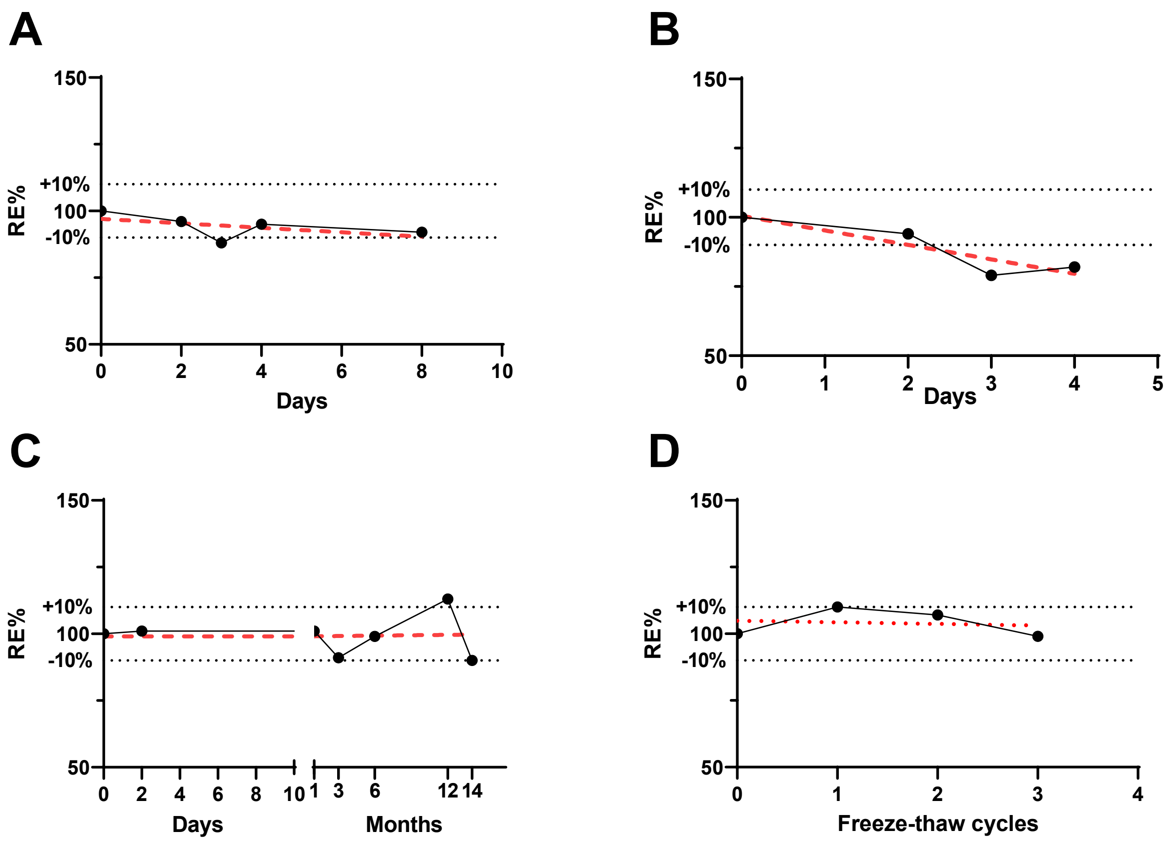

Fig. S1. Stability of PRO-C3 with storage and freeze-thaw. Serum samples from 10 NASH patients were measured within 4 hours of blood collection in order to determine PRO-C3 reference value (i.e. fresh sample). Samples were aliquoted and stored at (A) 8 °C, (B) 25 °C and (C) -80 °C and measured at the following time points after storage: 48 hours (8 °C and 25 °C storage), 72 hours (8 °C and 25 °C storage), 8 days (8 °C storage), 1 month, 3 months, 6 months, 12 months and 24 months (-80 °C storage). (D) Freeze-thaw stability of the analyte was also assessed up to 3 freeze-thaw cycles, where samples were repeatedly stored at -80 °C. The time period between each freeze-thaw cycle was at least 24 hours. Acceptance criteria for analyte stability was defined as ≤10% change from

fresh sample for each time point and a weighted Deeming slope of 1.0±0 when regressing the measurement from each condition to the fresh sample measurement.

Table S1. Assessing the precision of the PRO-C3 ELISA

Sample ID	Overall mean	Within run CV%	Lot CV%		Operator CV%		Total CV%
			LOT 1	LOT 2	Operator 1	Operator 2	
1	5.0	9.2	12.3	13.7	10.8	13.3	13.2
2	6.3	4.0	10.6	9.5	9.4	10.5	11.0
3	11.1	8.5	10.7	13.7	11.3	13.3	12.8
4	12.4	6.3	9.4	10.0	8.7	11.3	10.3
5	16.8	2.7	7.6	7.7	6.9	8.9	8.4
6	19.2	8.0	9.7	10.2	9.3	11.5	10.5

Supplemental Table 1. Assessing the precision of the PRO-C3 ELISA. Data presented are mean values or CV%. Sample 1 had a value below LLOQ (6.1ng/ml) and was therefore excluded from the study.

Table S2. Interference of endogenous and exogenous compounds on PRO-C3 levels

Interferent	Mean Sample 1 (Control) [ng/mL]	Mean Sample 1 (Test) [ng/mL]	Interference [%]	Mean Sample 2 (Control) [ng/mL]	Mean Sample 2 (Test) [ng/mL]	Interference [%]
Bilirubin Unconjugated	12.9	12.4	-3.9	24.6	23.4	-4.9
Bilirubin Conjugated	12.7	12.1	-4.7	24.5	24.2	-1.2
Hemoglobin	11.7	12.2	4.3	21.6	22.1	2.3
Biotin	11.4	11.0	-3.5	23.7	24.2	2.1
Intralipid	13.4	13.0	-3.0	24.9	24.7	-0.8
Human Serum Albumin	12.8	13.9	8.6	22.0	22.5	2.3
RF	12.8	11.5	-10.2	23.0	24.6	7.0
HAMA*	11.3	11.5	1.8	23.3	22.8	-2.1
Human IgG	13.4	14.1	5.2	26.8	25.5	-4.9
PEG-Fgf21	10.5	9.8	-6.7	21.1	20.5	-2.8
Metformin	11.6	11.3	-2.6	22.2	21.1	-5.0
Omeprazole	12.0	12.4	3.3	21.9	22.2	1.4
Levothyroxine	11.6	11.0	-5.2	24.5	23.7	-3.3
Simvastatin	10.4	10.0	-3.8	20.7	20.3	-1.9
Cyclobenzaprine	12.1	13.1	8.3	23.4	22.9	-2.1
Hydrochlorothiazide	12.7	13.2	3.9	25.2	26.0	3.2
Lisinopril	14.1	14.0	-0.7	26.0	27.5	5.8
Fluticasone	10.1	11.0	8.9	18.3	19.6	7.1
Ciprofloxacin*	14.1	13.0	-7.8	22.6	23.2	2.7
Acetaminophen	13.8	14.6	5.8	26.0	24.7	-5.0

Table S2. Interference of endogenous and exogenous compounds on PRO-C3 levels. Data are reported as mean percentage interference. Test samples are spiked with the interferent dissolved in a dissolvent at the concentration listed in Table 1 (Main text). Control samples are spiked with an equivalent volume of dissolvent used for the interferent. * HAMA and Ciprofloxacin were subjected to dose-series testing but showed no interference above the predefined acceptance criteria in all concentrations.

Table S3. Demographic data from patients from Mainz and the Nottingham cohorts

	Mainz (N=139)	Nottingham (N=83)	Total (N=222)	p value
**Age**				< 0.001
Median (Q1, Q3)	53.0 (43.0, 59.5)	60.0 (52.9, 68.2)	56.0 (46.6, 63.3)	
**Gender**				0.782
Female	66 (47.5%)	41 (49.4%)	107 (48.2%)	
Male	73 (52.5%)	42 (50.6%)	115 (51.8%)	
**BMI**				0.058
Median (Q1, Q3)	32.2 (29.2, 36.2)	34.0 (30.0, 38.0)	33.0 (29.5, 37.0)	
**AST**				0.275
Median (Q1, Q3)	50.0 (38.0, 67.0)	46.0 (33.0, 66.0)	48.5 (36.0, 67.0)	
**ALT**				< 0.001
Median (Q1, Q3)	74.0 (51.5, 114.5)	49.0 (30.2, 69.0)	64.0 (41.0, 95.0)	
**Bilirubin**				< 0.001
Median (Q1, Q3)	0.7 (0.5, 0.9)	11.0 (8.0, 14.0)	1.0 (0.6, 9.0)	
**Platelets**				0.249
Median (Q1, Q3)	231.0 (186.5, 277.5)	213.0 (175.5, 270.5)	227.5 (181.0, 275.8)	
**T2DB**				0.009
0	83 (60.1%)	34 (42.0%)	117 (53.4%)	
1	55 (39.9%)	47 (58.0%)	102 (46.6%)	
**Hypertension**				< 0.001
0	37 (26.8%)	42 (50.6%)	79 (35.7%)	
1	101 (73.2%)	41 (49.4%)	142 (64.3%)	
**NAS**				0.008
0	4 (2.9%)	2 (2.4%)	6 (2.7%)	
1	13 (9.4%)	7 (8.4%)	20 (9.0%)	
2	16 (11.5%)	3 (3.6%)	19 (8.6%)	
3	25 (18.0%)	9 (10.8%)	34 (15.3%)	
4	45 (32.4%)	18 (21.7%)	63 (28.4%)	
5	27 (19.4%)	28 (33.7%)	55 (24.8%)	
6	6 (4.3%)	10 (12.0%)	16 (7.2%)	
7	3 (2.2%)	4 (4.8%)	7 (3.2%)	
8	0 (0.0%)	2 (2.4%)	2 (0.9%)	
**Steatosis**				< 0.001
0	8 (5.8%)	4 (4.8%)	12 (5.4%)	
1	49 (35.3%)	20 (24.1%)	69 (31.1%)	
2	70 (50.4%)	13 (15.7%)	83 (37.4%)	
3	12 (8.6%)	46 (55.4%)	58 (26.1%)	
**Lobular Infl.**				0.272
0	33 (24.3%)	16 (19.3%)	49 (22.4%)	
1	78 (57.4%)	58 (69.9%)	136 (62.1%)	
2	21 (15.4%)	7 (8.4%)	28 (12.8%)	

3	4 (2.9%)	2 (2.4%)	6 (2.7%)	
**Ballooning**				0.052
0	24 (17.3%)	12 (14.5%)	36 (16.2%)	
1	95 (68.3%)	48 (57.8%)	143 (64.4%)	
2	20 (14.4%)	23 (27.7%)	43 (19.4%)	
**Fibrosis**				< 0.001
F0/F1	40 (28.8%)	17 (20.5%)	57 (25.7%)	
F2	51 (36.7%)	8 (9.6%)	59 (26.6%)	
F3	32 (23.0%)	34 (41.0%)	66 (29.7%)	
F4	16 (11.5%)	24 (28.9%)	40 (18.0%)	
**PROC3**				0.029
Median (Q1, Q3)	11.4 (9.4, 16.4)	13.8 (10.9, 17.4)	12.6 (9.8, 16.8)	

Table S3. Demographic data from patients from Mainz and the Nottingham cohorts.

To test for differences between the two cohorts and the combined cohort, we used a Kruskal-Wallis test.

Supplementary references

1. EMA. Guideline on bioanalytical method validation. *EMEA/CHMP/EWP/192217/2009 Rev. 1 Corr. 2*** **44**, 1–23 (2012).

2. *CLSI EP25-A - Evaluation of Stability of In Vitro Diagnostic Reagents*. (Clinical And Laboratory Standards Institute [CLSI], 2009).

3. *BS EN ISO 23640:2015*. (2015).

4. *CLSI EP07-A2 - Interference Testing in Clinical Chemistry; Approved Guideline - Second Edition*. (Clinical And Laboratory Standards Institute [CLSI], 2005).

5. *CLSI EP05-A3 - Evaluation of Precision of Quantitative Measurement Procedures*. (Clinical And Laboratory Standards Institute [CLSI], 2014).

6. Labenz, C., Huber, Y., Kalliga, E., Nagel, M., Ruckes, C., Straub, B. K., *et al.* Predictors of advanced fibrosis in non-cirrhotic non-alcoholic fatty liver disease in Germany. *Aliment. Pharmacol. Ther.* **48**, 1109–1116 (2018).

7. Daniels, S. J., Leeming, D. J., Eslam, M., Hashem, A. M., Nielsen, M. J., Krag, A., *et al.* ADAPT: An Algorithm Incorporating PRO-C3 Accurately Identifies Patients With NAFLD and Advanced Fibrosis. *Hepatology* **69**, 1075–1086 (2019).

8. Kleiner, D. E., Brunt, E. M., Van Natta, M., Behling, C., Contos, M. J., Cummings, O. W., *et al.* Design and validation of a histological scoring system for nonalcoholic fatty liver disease. *Hepatology* **41**, 1313–1321 (2005).
