# Supplementary material for: Determining a healthy reference range and factors potentially influencing PRO-C3 – A biomarker of liver fibrosis
Source: JHEP Rep. 2021 Jul 10;3(4):100317. doi: 10.1016/j.jhepr.2021.100317 (PMC8385245; doi:10.1016/j.jhepr.2021.100317)
Supplement: Multimedia component 2 [file mmc2.pdf]

JOURNAL
OF HEPATOLOGY

Journal of Hepatology

CTAT methods

Tables for a "Complete, Transparent, Accurate and Timely account" (CTAT) are now mandatory for all revised submissions. The aim is to enhance the reproducibility of methods.

- Only include the parts relevant to your study
- Refer to the CTAT in the main text as 'Supplementary CTAT Table'
- Do not add subheadings
- Add as many rows as needed to include all information
- Only include one item per row

If the CTAT form is not relevant to your study, please outline the reasons why:

1.1 Antibodies

Name	Citation	Supplier	Cat no.	Clone no.

1.2 Cell lines

Name	Citation	Supplier	Cat no.	Passage no.	Authentication test method

1.3 Organisms

Name	Citation	Supplier	Strain	Sex	Age	Overall n number

1.4 Sequence based reagents

Name	Sequence	Supplier

1.5 Biological samples

Description	Source	Identifier
NAFLD patients (DE)	Department of Internal Medicine I, University Medical Centre of the Johannes Gutenberg-University, Mainz, Germany.	N/A
NAFLD patients (DK)	Department of Clinical Medicine, Faculty of Health and Medical	N/A

	**Sciences, University of Copenhagen, Copenhagen Denmark<br>Gastro Unit, Medical Division, Hvidovre. Hospital, University of Copenhagen, Hvidovre, Denmark.**	
**NAFLD (UK)**	**Nottingham Digestive Diseases Centre, University of Nottingham, Nottingham, United Kingdom.<br>NIHR Nottingham Biomedical Research Centre at the Nottingham University Hospitals NHS Trust and the University of Nottingham, Nottingham, United Kingdom.**	**N/A**

1.6 Deposited data

**Name of repository**	**Identifier**	**Link**
**Healthy volunteers**	**Discovery Life Sciences**	**info@dls.com**
**NASH patients**	**Reprocell**	**https://www.reprocell.com/contact**

1.7 Software

**Software name**	**Manufacturer**	**Version**

1.8 Other (e.g. drugs, proteins, vectors etc.)

**PRO-C3 ELISA analysis of**	**Nordic Bioscience**	**Lot. No.**
Technical testing (CLSI)	Cat.no. 1700-03	EB1806A<br>EB1807A<br>EB1808A
Healthy volunteers	Cat.no. 1700-03	EB1807A
Reprocell	Cat.no. 1700-03	EB1807A
NAFLD/NASH : UK, DK, DE (for full adresse see above)	Cat.no. 1700-03	EB1911A<br>EB1808A

1.9 Please provide the details of the corresponding methods author for the manuscript:

Daniel Guldager Kring Rasmussen, MSc, PhD
E-mail: dgr@nordicbio.com
Tel.No.: +4544525252

Nordic Bioscience<br>Herlev Hovedgade 205-207, DK-2730, Denmark

2.0 Please confirm for randomised controlled trials all versions of the clinical protocol are included in the submission. These will be published online as supplementary information.

